# Viability of Self‐Taken Vaginal Swab Samples for RNA‐Based Biomarker Analysis in Cervical Disease

**DOI:** 10.1002/jmv.70737

**Published:** 2025-12-08

**Authors:** Harry Scott, Andrew Stevenson, Daniel Mair, William M. Rooney, Hana McMahon, Laila Sara Arroyo Mühr, Kate Cuschieri, Sheila V. Graham

**Affiliations:** ^1^ MRC‐University of Glasgow Centre for Virus Research, School of Infection and Immunity, College of Medical Veterinary and Life Sciences, University of Glasgow Garscube Estate Glasgow UK; ^2^ Institute for Regeneration and Repair University of Edinburgh Edinburgh UK; ^3^ Center for Cervical Cancer Elimination, Department of Clinical Science, Intervention and Technology, Karolinska Institutet F56 Karolinska University Hospital Stockholm Sweden; ^4^ Scottish HPV Reference Laboratory, Royal Infirmary of Edinburgh 51 Little France Crescent NHS Lothian Edinburgh UK

**Keywords:** cervical screening, human papillomavirus, RNA quality biomarkers, self taken samples

## Abstract

High risk human papillomaviruses (hrHPVs) cause most cervical cancers. Cervical screening programmes aim to identify precancerous disease using detection of hrHPV nucleic acid using clinician‐taken liquid‐based cytology (LBC) samples, or increasingly, self‐taken samples (STSs). However, STSs are incompatible with traditional cytology triage. Therefore, development of novel triage tests is essential. Quantification of cellular and viral mRNA biomarkers is one option, but little is known about mRNA quality in STSs. We extracted RNA from two sets of STSs (reflecting separate sampling devices) from the Scottish HPV Archive (SHA) and the Swedish Cervical Cytology Biobank (SCCB). We investigated whether the *ACTB*, *GAPDH* and *p16* RNAs could be amplified by reverse transcription quantitative PCR (RT‐qPCR). RNA was degraded in both sets of samples, but samples from the Scottish HPV Archive were generally suitable for RT‐qPCR analysis, while samples from the SCCB were mostly unsuitable. Genomic DNA contamination was detected in 11.6% of samples. The quantity and quality of RNA derived from STSs was unaffected by storage of the original sample at −70°C for a period of 1 year. These data suggest that feasibility of utilising STSs for mRNA expression work is device‐dependent and that optimisation of collection and storage systems is warranted.

**Abbreviations**
ACTB: Beta (β)‐actin
CIN1: Cervical Intraepithelial Neoplasia Grade 1
Ct: cycle threshold
DMEM: Dulbecco's Modified Eagle Medium
GAPDH: glyceraldehyde‐3‐phosphate dehydrogenase
gDNA: genomic DNA
hrHPV: High‐risk human papillomavirus
LBC: Liquid‐based cytology
p16: p16INK4A
PBS: Phosphate buffered saline
RIN: RNA Integrity Number
RT‐qPCR: Reverse Transcription quantitative PCR
SCCB: Swedish Cervical Cytology Biobank

## 1 Introduction

Cervical cancer is caused by “high risk” genotypes of human papillomavirus (hrHPV). Cervical screening programmes aim to detect precancerous disease, usually through molecular testing for hrHPV nucleic acid (NA). This type of HPV based screening can be performed on both clinician‐taken liquid based cytology (LBC; smear) samples and on self‐taken samples. Self‐sampling can mitigate barriers to clinician‐based screening and is associated with reduced material and time costs for healthcare services [1, 2]. Additionally, the availability of self‐sampling has the potential to enhance engagement in underserved populations who have traditionally had lower engagement with cervical screening and are therefore most at risk of cervical disease/cancer [3, 4]. Moreover, HPV testing on self‐taken samples shows similar performance to clinician‐taken samples for the detection of high‐grade cervical disease [5]. Thus, the pace of implementation of self‐sampling as a general option to all, or for underserved individuals is rapid [6]. However, the way self‐sampling is offered differs according to setting. Additionally, a variety of sampling devices and associated HPV tests have been implemented. Notwithstanding this variety one common challenge is that due to the small proportion of cervical epithelial cells in self‐taken samples, cytological evaluation is unfeasible [7]. This means that women who test positive for HPV NA by self‐sampling are generally required to attend a clinic for a follow‐up clinician‐taken sample to be collected on which cytology can be performed, as is the case in Australia [8]. The risk created by this is that some women will not attend the follow‐up appointment, particularly those who were originally hesitant to engage in the screening process [7].

The development of specific biomarkers is needed as alternatives to cytology to support the risk stratification of primary HPV infection using LBC/self‐taken samples. Such tests would minimise loss to follow‐up and speed up the referral process. Tests which investigate the methylation of specific cellular genes are arguably the front‐runners [9, 10, 11]. However, real‐time quantitative PCR (qPCR) assays which investigate expression levels (mRNA levels) of genes associated with disease could be advantageous due to the practical ease of use, and the fact that qPCR systems are in abundance in clinical service laboratories [12].

There is currently little information on the suitability of routinely taken vaginal self‐samples for mRNA expression work and how this may differ according to sampling device. Most countries that have implemented self‐sampling have relied on HPV DNA based tests and while DNA is highly stable it is notable that sampling device‐driven differences in performance have been observed [13]. Biobanks serve as important resources for the evaluation of new biomarkers and those which contain residual self‐taken samples previously annotated for HPV and disease status have value in this regard. Here, we undertook a feasibility study to investigate the quantity and quality of RNA extracted from residual self‐taken vaginal swab samples (reflecting two devices) obtained from two national biobanks; the Scottish HPV Archive and the Swedish Cervical Cytology Biobank. RNA extracted from the Scottish HPV Archive (SHA) samples was suitable for analysis by RT‐qPCR, with expression of reference genes beta‐actin (*ACTB)* and *GAPDH* being detected in all samples, while a candidate biomarker, *p16* RNA was detected in 82.1% of samples. In contrast, a cohort of samples obtained from the Swedish Cervical Cytology Biobank (SCCB) [14] yielded lower quality RNA and showed reduced performance in RT‐qPCR analysis. The data suggests that differences in sample collection and/or storage may be important in determining the suitability of biobanked samples for mRNA‐based testing.

## 2 Methods

### 2.1 Ethics

The Scottish HPV Archive (SHA) is a biobank that comes under the auspices of NHS Lothian National Research for Scotland bioresource (REC 25/ES/0030). Self‐taken vaginal swab samples from the SHA were obtained through an application to the SHA Steering Group (application reference SA‐0069).

Collection of Swedish Cervical Cytology Biobank (SCCB) samples and use for biological research was approved by the Regional Ethical Review Board in Stockholm (decision number 2014/1242‐31/4).

### 2.2 Cell Culture

NIKS (HPV‐negative human normal immortalised keratinocytes) [15] and W12E (HPV16‐infected human cervical epithelial cells established from a low‐grade lesion histologically graded CIN1) [16, 17] cells were cocultured in E‐medium with mitomycin C‐treated J2 3T3 fibroblast feeder cells as previously described [16]. Differentiation was induced by growth to high density in 1.2 mM Ca^2+^. 3T3 cells were grown in Dulbecco's modified Eagle medium (DMEM) with 10% donor calf serum. Before harvesting, 3T3 cells were removed by trypsinization, and cell layers were washed twice with phosphate‐buffered saline (PBS). Cells were maintained in 5% CO_2_ at 37°C.

### 2.3 HPV Testing

HPV testing for the SHA samples used the Aptima HR HPV test (Hologic Inc, Belgium). 45 samples were HPV‐positive and 50 samples were HPV‐negative. HPV‐positive samples were then tested using an Allplex HPV HR Detection assay (Seegene, South Korea) to give individual results for 14 high‐risk HPV genotypes (Supporting information: Table S1). Five samples that returned a positive Aptima result were negative for all 14 types tested by Allplex. Disease status was available for all HPV‐positive samples, with 27 samples being associated with no disease (< = CIN1 or no biopsy indicated at colposcopy) and 18 samples being associated with low‐grade disease (CIN1). HPV testing for the SCCB used the cobas HPV test (Roche, Switzerland). All 19 SCCB samples were HPV‐positive. No information on disease status was provided for SCCB samples.

### 2.4 RNA Extraction, Quality and Quantity Testing

For samples from the SHA, RNA was extracted from a sample volume of 400 μL using a KingFisher Flex Purification System (ThermoFisher Scientific) and an RNAdvance Blood Kit (Beckman Coulter). Steps were as described in the kit instructions except that 30 μL of proteinase K was added to each sample. DNase treatment was 10 U of Ambion DNase I (ThermoFisher Scientific) and incubation at 37°C for 15 min. RNA was eluted in 25 μL RNase free H_2_O. An extraction blank containing RNase free H_2_O was included in each extraction plate. RNA quantity was measured by Nanodrop and Qubit RNA High Sensitivity assay (ThermoFisher Scientific). RNA quality was assessed using a 4200 Tapestation system (Agilent). Samples were also tested by Qubit dsDNA High Sensitivity assay (ThermoFisher Scientific). Extracted RNA was stored at −70°C. For samples from the SCCB, 300 μL of RNase free H_2_O was added to 100 μL of each sample and RNA was extracted and quality‐assessed as above.

### 2.5 DNA Gel Electrophoresis and Gel Extraction

Agarose gels (0.7%) were electrophoresed at 60 V for 45 min, stained with ethidium bromide (final concentration 0.2 µg/mL) and imaged using a UV transilluminator. Gel fragments were excised and DNA was purified using a QIAquick gel extraction kit (Qiagen).

### 2.6 cDNA Synthesis

cDNA was synthesized using a Maxima First Strand cDNA synthesis kit (Thermo Fisher Scientific), with additional dsDNase treatment, following the manufacturer's instructions. 8 μL of RNA was used for each cDNA reaction. Reverse transcriptase negative reactions were included where RNase free H_2_O was added in place of enzyme mix. cDNA was diluted to a final volume of 65 μL using RNase free H_2_O and stored at −20°C until use.

### 2.7 qPCR

Triplex qPCR reactions included two reference genes (*ACTB* and *GAPDH*) and one target gene (*p16* ^*INK4A*^, subsequently referred to as *p16*). Primers and probes were designed using IDT's Primer Quest Tool and purchased from Eurogentec (Eurogentec, Belgium) (https://eu.idtdna.com/PrimerQuest/Home/Index). Primer/probe sequences for *ACTB* were: forward primer: 5’‐AGCGCGGCTACAGCTTCA‐3’, reverse primer: 5’‐CGTAGCACAGCTTCTCCTTAATGTC‐3’, probe: 5’‐HEX‐ATTTCCCGCTCGGCCGTGGT‐BHQ‐1‐3’. Primer/probe sequences for *GAPDH* were: forward primer: 5’‐GGTGTGAACCATGAGAAGTATGA‐3’, reverse primer: 5’‐GAGTCCTTCCACGATACCAAAG‐3’, probe: 5’‐Cy5‐AGATCATCAGCAATGCCTCCTGCA‐BHQ‐2‐3’. Primer/probe sequences for *p16* were: forward primer: 5’‐GCACATTCATGTGGGCATTT‐3’, reverse primer: 5’‐GACTCAAGAGAAGCCAGTAACC‐3’, probe: 5’−6‐FAM‐CCGGAAGCTGTCGACTTCATGACA‐BHQ‐1‐3’. Amplicon lengths were 72 bp for ACTB, 215 bp for GAPDH and 114 bp for p16. Reaction efficiencies in triplex reactions were determined by dilution series using NIKS cDNA. Efficiencies were 92% for *ACTB*, 89% for *GAPDH* and 98% for *p16*.

Triplicate qPCR reactions contained 1xTakyon Low ROX Probe MasterMix dTTP blue (Eurogentec), 5 μL of cDNA, 150 nM of primers for each reference gene plus 900 nM primers for *p16* and 100 nM of probes in a volume of 20 μL. No template control reactions and reactions containing W12 cell line cDNA were included on each plate as negative and positive controls respectively. Reverse transcriptase‐negative reactions were also included in each plate. Reactions were run using an ABI7500 Fast RT‐qPCR machine (Applied Biosystems, USA). qPCR conditions were: 1 cycle of 50°C for 2 min then 95°C for 3 min, followed by 40 cycles of 95°C for 10 s, 60°C for 1 min.

### 2.8 Data Analysis

Tapestation data was analysed using Tapestation Analysis Software v5.1 (Agilent). RT‐qPCR data was analysed using ABI 7500 Software v2.3 (Applied Biosystems). To determine cycle threshold (Ct) values, manual thresholds of 0.15 and 0.2 were set for *ACTB*/*GAPDH* and *p16* respectively. Data were exported to Microsoft Excel for relative expression calculations. Samples where *p16* was not detected in all three technical replicates were excluded from further analysis. Relative *p16* expression values were calculated with the Pfaffl method [18] using W12 cell line cDNA as the calibrator sample. Figures were produced using Graphpad Prism v.9.2.0.

### 2.9 Statistical Analysis

All statistical analysis was performed using Graphpad Prism v9.2.0. Before analysis, the distributions of the groups to be compared were assessed by Shapiro‐Wilk test. As data were not normally distributed, comparison between groups was carried out using a Mann‐Whitney test (for comparisons of HPV‐positive and HPV‐negative samples, comparison of relative p16 expression and comparisons of RNA quantity/quality before and after 1 year of original sample storage at −70°C). Correlations between reference gene Ct values and RNA concentration (measured by Qubit) were assessed using a Spearman's rank correlation test.

## 3 Results

### 3.1 Scottish HPV Archive (SHA) Sample Cohort

Self‐taken vaginal swab samples [95] were obtained from the Scottish HPV Archive (SHA). All samples were residual volumes from a previous study on self‐sampling in women who had defaulted from cervical screening in a single health board in Scotland [18]. Sample kits were mailed to participants at home between August 2020 and August 2021. Samples were collected by participants using a Multitest specimen kit (Hologic Inc, Belgium). These were stored in 2.9 mL of Specimen Transport Media (Hologic Inc, Belgium) at −20°C for around a year (the duration of the study) before being moved to longer term archival storage at −80°C. Aliquots of approximately 2 ml per sample were shipped to the research laboratory and stored at −70°C before RNA extraction. 45/95 samples were HPV‐positive. 27 HPV‐positive samples were associated with no disease (≤ CIN1 or no biopsy indicated at colposcopy) and 18 samples being associated with low‐grade disease (CIN1).

### 3.2 RNA Extraction Yields Quantifiable RNA From Biobanked Self‐Taken Samples

**Figure 1:**
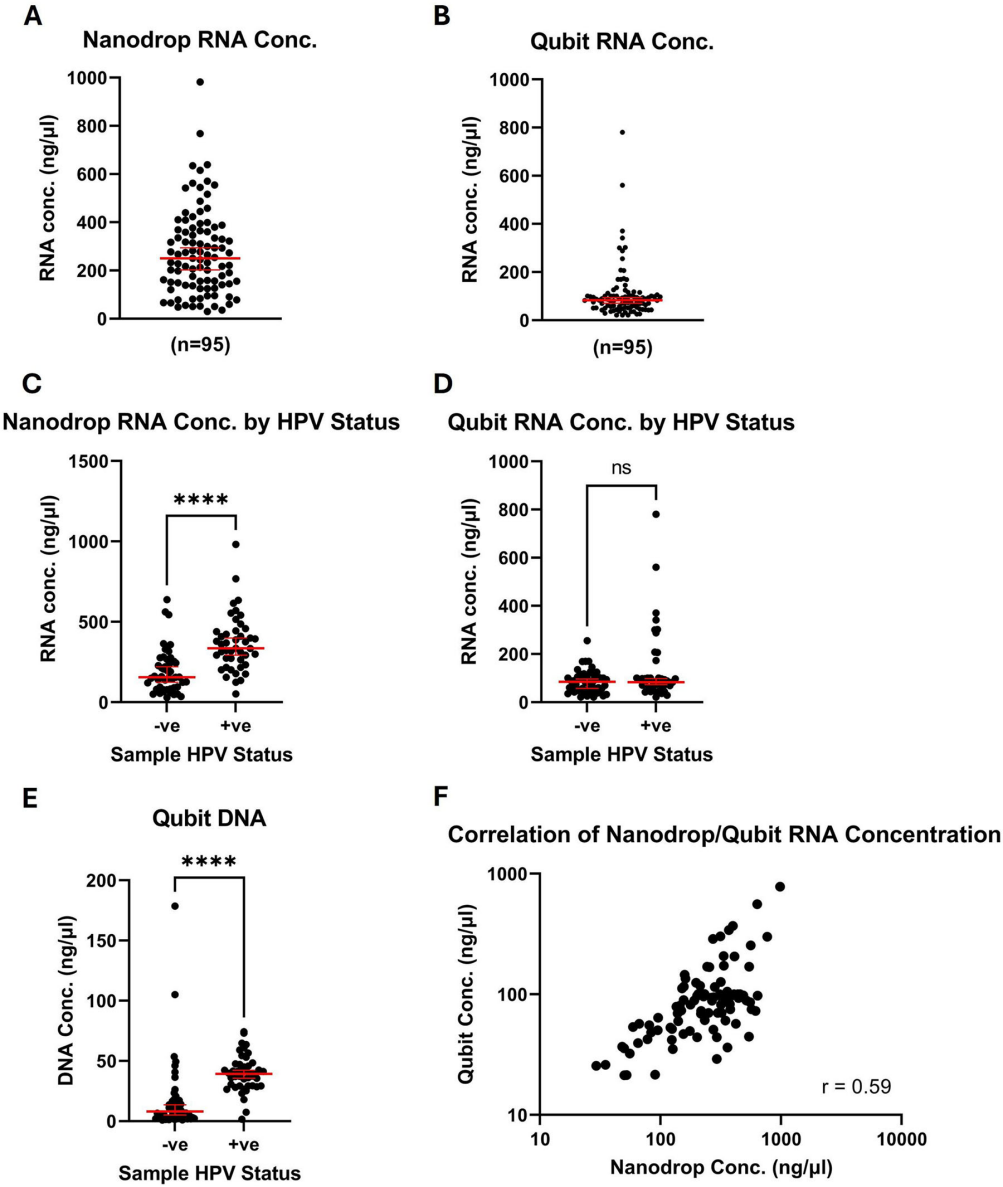
Quantity of RNA and Residual DNA Extracted from Self‐taken Vaginal Swab Samples. Concentrations of extracted RNA were determined by Nanodrop (A) and Qubit (B). Extracted RNA was compared between HPV‐negative and HPV‐positive samples using Nanodrop (C) and Qubit (D) readings. Concentrations of residual DNA were also assessed for each sample by Qubit (E). Red lines = median and 95% CI of each group, **** = *p* < 0.0001 (Mann‐Whitney test). The correlation between Nanodrop and Qubit RNA concentration was also assessed (F), r = Spearman's correlation coefficient.

RNA concentrations were measured by both Nanodrop and Qubit. Nanodrop uses UV spectrophotometry to measure nucleic acid concentrations, while Qubit is a fluorometer and quantifies dyes which specifically bind to either RNA or DNA. Therefore, since both RNA and DNA have a maximum absorbance at a wavelength of 260 nm, Nanodrop measures all nucleic acids, while Qubit measures either DNA or RNA concentration. Median RNA/DNA concentrations and ranges are shown for each quantification method in Table 1. The overall median RNA concentration was 251 ng/μL by Nanodrop testing and 83 ng/μL by Qubit RNA testing (Figure 1A–B). Nanodrop quantification indicated that HPV‐positive samples had a significantly higher concentration of RNA than HPV‐negative samples (Figure 1C). There was no difference between RNA concentrations of HPV‐negative and HPV‐positive samples as determined by Qubit (Figure 1D). However, HPV‐positive samples had significantly higher levels of DNA contamination, as measured by a Qubit DNA quantification assay, following RNA extraction (Figure 1E), which would contribute to higher Nanodrop readings. Despite the differences in quantification method, there was a moderately strong correlation between Nanodrop and Qubit RNA readings (Spearman coefficient *r* = 0.59) (Figure 1F). Overall, levels of RNA suitable for RT‐qPCR experiments were derived from every sample in the SHA cohort.

**Table 1:** Comparison of Median Nucleic Acid Quantification Measurements in HPV‐negative and HPV‐positive Samples from the SHA.

HPV status	Nanodrop RNA (ng/µL)	Qubit RNA (ng/µL)	Qubit DNA (ng/µL)
HPV‐negative (*n* = 50)	**156** (29–638)	**84** (21–255)	**8** (1–179)
HPV‐positive (*n* = 45)	**335** (52–982)	**83** (21–780)	**39** (2–61)
All Samples (*n* = 95)	**251** (29–982)	**83** (21–780)	**26** (1–179)

*Note:* Range is shown in brackets.

### 3.3 RNA Extracted From Self‐Taken Samples Is Generally Degraded

**Figure 2:**
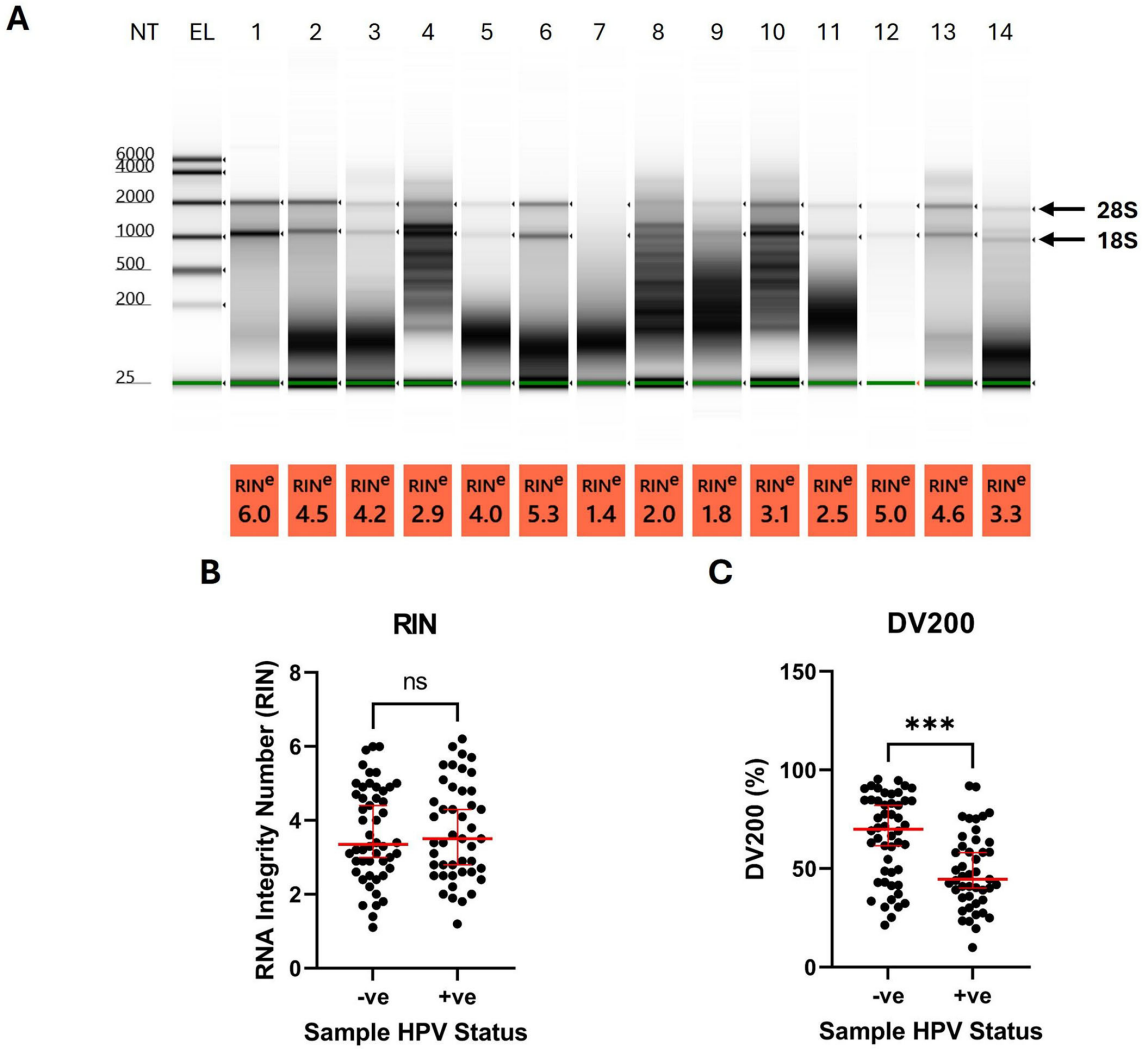
Tapestation Analysis for Quality Testing of RNA Extracted from Self‐taken Vaginal Swab Samples. (A) Tapestation gel showing electrophoresis results for each sample and associated RIN values below each lane. 18S and 28S ribosomal RNA bands are indicated by arrows on the right‐hand side of the gel. EL = Electronic RNA ladder. Ribosomal RNA bands were absent in some samples e.g. lane 7. (B) RNA integrity number (RIN) of each sample separated by sample HPV status. RIN represents the quality of RNA derived from each sample, with high RIN values corresponding to higher quality RNA. (C) DV200 values of each sample separated by sample HPV status. DV200 represents the percentage of fragments which are 200‐10,000 nucleotides in length, with higher percentages corresponding to higher quality RNA. Red lines = median and 95% CI of each group, *** = *p* < 0.001. Distribution of the data were assessed using a Shapiro‐Wilk test which showed that data were not normally distributed, therefore statistical significance was assessed using a Mann‐Whitney test.

RNA quality was analysed using an Agilent Tapestation: an electrophoretic technique that gives the mass distribution of the RNA samples (Figure 2A). Based on these data, RNA Integrity Number (RIN) and a DV200 value are determined for each sample. RIN values range from 1 (lowest quality RNA) to 10 (highest quality RNA). They are calculated based on features of the electropherogram, including the 18S/28S ribosomal subunit bands and the fast area ratio, a measure of the proportion of RNA between the 5S and 18S ribosomal peaks [19]. DV200 values represent the percentage of fragments between 200 and 10,000 nucleotides in length, with values above 70% indicating less heavily degraded RNA which is more suitable for downstream molecular analysis such as qPCR and sequencing. Values of 50%–70% represent moderately degraded RNA which may still be of acceptable quality for these purposes [20, 21].

RIN values were similar between HPV‐positive and HPV‐negative samples, with medians of 3.5 and 3.4 respectively and an overall range of 1.1–6.2 (Figure 2B). This indicates that RNA derived from these samples is generally degraded. However, although 18S and 28S ribosomal RNA peaks were difficult to distinguish or missing entirely for some samples (e.g. lanes 7, 12), many of the samples displayed intact ribosomal RNA. 18S and 28S ribosomal bands (arrowheads on the right‐hand side of the electropherogram) were below the expected size in this analysis, with sizes of ~1 kb and ~2 kb respectively compared to expected sizes of 2 kb and 5 kb. Despite this, electrophoretic mobility of ribosomal RNA bands was consistent. This aberrant sizing effect was likely caused by the use of the internal electronic ladder. Therefore, in cases where ribosomal RNA peaks were incorrectly assigned by the software, these were manually adjusted. Due to all RNAs being sized as smaller than expected, DV200 values of all samples are likely to be underestimated. This Tapestation mis‐sizing effect has also been observed previously in analysis of clinician‐taken samples [22]. While RIN values were consistent across samples regardless of HPV status, DV200 values were significantly higher in HPV‐negative samples than HPV‐positive samples (70% vs. 45%) (Figure 2C). This suggests that RNA produced from HPV‐negative samples is likely to be less heavily degraded and more suitable for downstream molecular analysis.

A subset of samples analysed by Tapestation (11/95, or 11.6% of samples) contained large fragments near the top of the electropherograms; likely DNA which was not fully degraded by DNase treatment during RNA extraction (Supporting information: Figure S1). Contaminated samples had a higher average DNA to RNA ratio than uncontaminated samples (0.66 *vs.* 0.30). To investigate this contamination further, DNA was prepared from the large bands in the gel from three samples. DNA contamination could be genomic DNA (gDNA) that was not fully removed during RNA preparation or could be chromosomal DNA from commensal bacteria. Analysis by qPCR revealed that human *ACTB* and *GAPDH* gene sequences were readily detected in these samples, strongly suggesting that the contaminant was gDNA (Supporting information: Table S2). This suggests DNase treatment during RNA extraction was inadequate for a subset of biobanked samples. However, further DNase treatment of these samples to remove gDNA was unsuccessful. Therefore, these samples were excluded from further analysis.

### 3.4 Quantity and Quality of RNA are Preserved in Self‐Taken Samples Stored for 1 Year at −70°C

**Figure 3:**
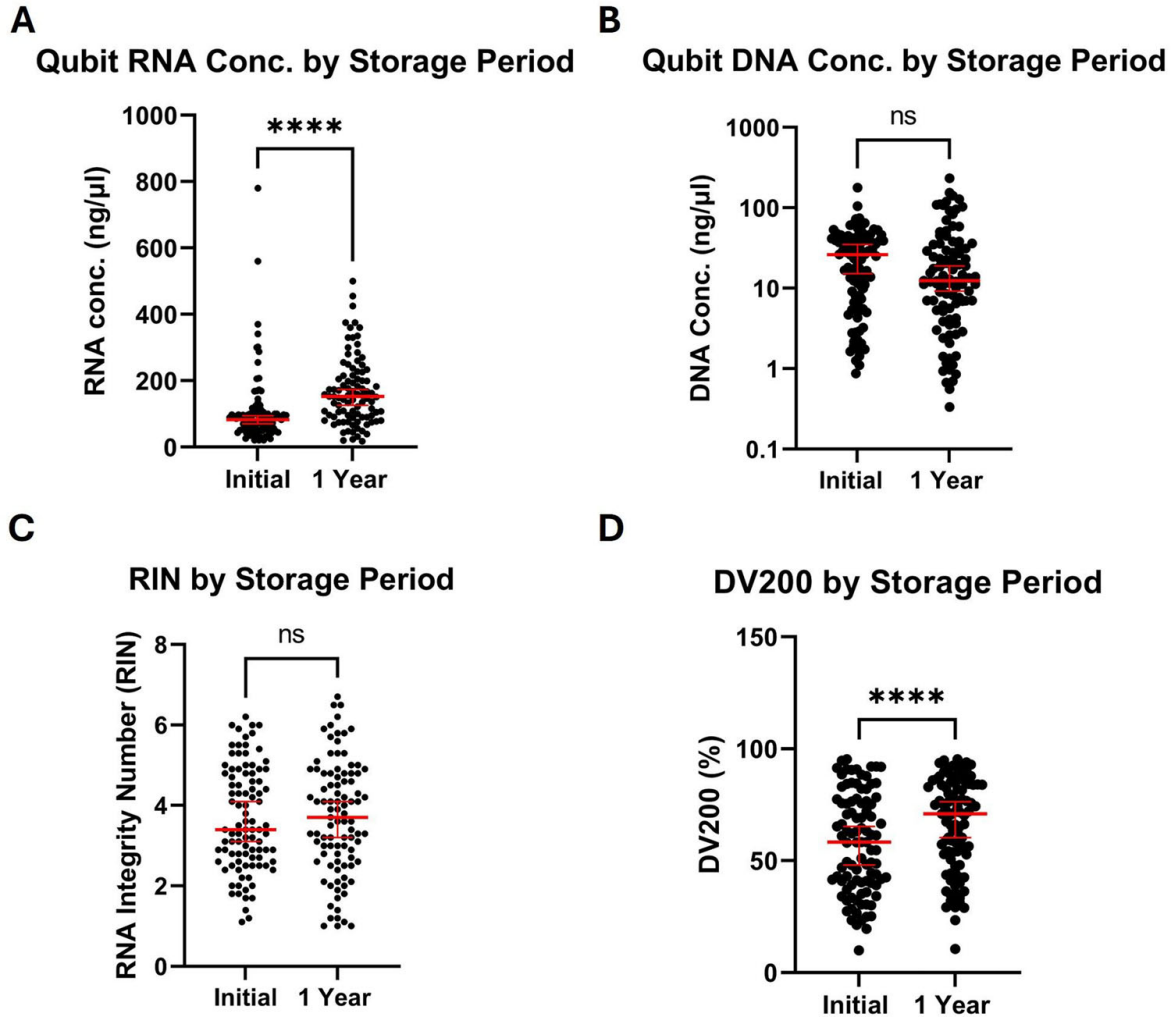
Comparison of RNA Quantity and Quality from 95 Biobank Samples Before and After Storage of Original Sample Material at −70°C for one year. Concentrations of RNA (A) and DNA (B) were determined by Qubit and compared before and after the storage period. RIN (C) and DV200 (D) values were also compared as before. Red lines = median and 95% CI of each group, **** = *p* < 0.0001, Mann‐Whitney test.

Next, we assessed the effect of long‐term storage of these samples on extracted RNA quantity and quality. Following initial RNA extraction, the remaining volume of each sample was stored at −70°C for 1 year before secondary RNA extraction and testing as above. Comparison of sample RNA quality and quantity should give a measure of any loss of integrity during storage of self‐taken swabs. Median nucleic acid concentrations and RNA quality measurements before and after sample storage are shown in Table 2. While the median concentration of Qubit‐measured RNA significantly increased in reextracted samples compared to initial extractions (Figure 3A), the average Qubit DNA concentration decreased (Figure 3B). RIN values of samples stored for 1 year were slightly higher than primary extracted samples, though this result was not statistically significant (Figure 3C). Similarly, DV200 values were significantly increased in samples extracted after 1 year of storage (Figure 3D), which is likely linked to the increased Qubit RNA concentrations of these samples. These results suggest that both RNA quality and quantity are preserved by storage of samples at −70°C for at least 1 year.

**Table 2:** Comparison of Median Nucleic Acid Quantity and Quality Measurements before and after 1 Year of SHA Samples Storage.

Measurement	Qubit RNA (ng/µL)	Qubit DNA (ng/µL)	RIN	DV200 (%)
Initial	**83** (21–780)	**26** (1–179)	**3.4** (1.1–6.2)	**58** (10–95)
1 Year reextraction	**153** (18–500)	**13** (0.3–234)	**3.7** (1–6.7)	**71** (23–95)

*Note:* Range is shown in brackets. RIN = RNA integrity number ranging from 1 (low quality RNA) to 10 (high quality RNA). DV200 represents the percentage of fragments 200–10,000 nucleotides in size.

Residual genomic DNA contamination was visibly present in 17/95 of the reextracted samples when analysed by Tapestation. Of these, seven samples were contaminated across the two sets of extractions, while the other ten were previously uncontaminated.

### 3.5 Common Reference Genes *ACTB* and *GAPDH* Are Readily Detected in Self‐Taken Samples

**Figure 4:**
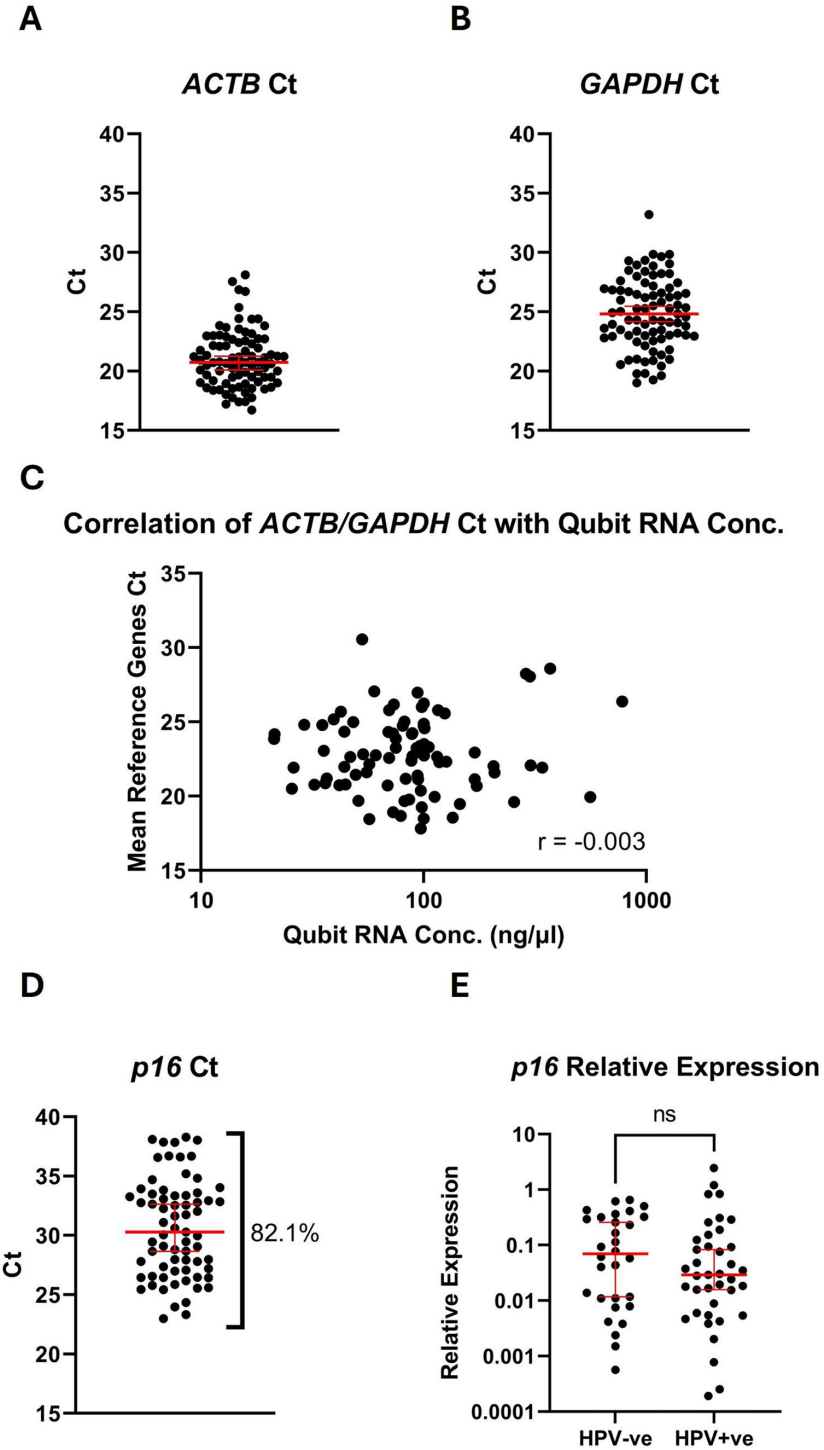
RT‐qPCR Testing of Self‐taken Swab Samples from the Scottish HPV Archive. Samples were analysed for expression of two reference genes: *ACTB* (A) and *GAPDH* (B). Each point represents an individual sample. *ACTB* and *GAPDH* were detected in all tested samples (*n* = 84). Ct = cycle threshold. (C). The correlation between RNA concentration measured by Qubit and reference genes Ct. Each point represents the geometric mean of the Cts for *ACTB* and *GAPDH* in an individual sample. Spearman's rank correlation test was performed to assess correlation (*r* = −0.003). (D) RT‐qPCR analysis (Ct values) of *p16* expression levels. (E) Relative *p16* expression levels in HPV‐negative and HPV‐positive cases. Relative expression of *p16* in each sample was calculated using the Pfaffl method. *n* = 30, 39 for HPV‐negative samples and HPV‐positive samples respectively. Red lines = median and 95% CI, ns = not significant, Mann‐Whitney test.

The suitability of SHA self‐taken samples for RT‐qPCR testing was assessed by detection of two common reference genes: *ACTB* and *GAPDH*. These genes have previously been shown to be stably expressed in liquid‐based cytology samples [23, 24]. *ACTB* and *GAPDH* expression was detected in all samples at a level below the Ct cutoff determined by RT‐negative reactions included in each plate (39 for *ACTB*, 40 for *GAPDH*) (Figure 4A–B). There was no correlation between RNA concentration (as measured by Qubit) and the geometric mean of the two reference gene Cts (Spearman coefficient *r* = −0.003), suggesting RNA concentration in this cohort was a poor predictor of reference gene expression (Figure 4C).

### 3.6 Prospective mRNA Biomarker *p16* Is Detected in the Majority of Self‐Taken Samples

To test the suitability of self‐taken samples for mRNA‐based detection of prospective target biomarkers we investigated expression levels of *p16*. As the current ‘gold‐standard’ biomarker for detection of HPV‐related cervical disease at the protein level [25, 26] and a target which has been shown to be upregulated at the mRNA level over different stages of cervical disease [22, 27], *p16* was a suitable target gene to investigate. Of the samples obtained from the SHA, *p16* was detected in 82.1% (69/84) below the RT‐qPCR cut‐off of Ct = 40 (Figure 4D). 15 samples were excluded due to undetermined Cts in at least one technical replicate. Comparison of samples by HPV status showed higher levels of *p16* in HPV‐negative samples than in HPV‐positive samples, though this difference was not statistically significant (Figure 4E).

### 3.7 Reduced RNA Yield From a Small Cohort of Swedish Cervical Cytology Biobank (SCCB) Samples

To investigate how samples from different biobanks may vary in their suitability for mRNA‐based testing, 19 self‐taken samples were obtained from the Swedish Cervical Cytology Biobank (SCCB). Samples were collected between 2020 and 2023 using a cobas PCR Media Dual Swab Sample Kit (Roche, Switzerland) and stored in 4.3 mL of cobas PCR Media (Roche, Switzerland). All samples were stored at −25°C. Aliquots of 100 μL per sample were shipped and stored at −70°C before RNA extraction. All 19 samples were HPV‐positive. Information on disease status was not provided for these samples.

RNA concentration in the SCCB sample group was much lower than the cohort of samples from the SHA, with a median RNA concentration of 11 ng/μL by Nanodrop testing and 2 ng/μL by Qubit testing (Table 3). DNA concentration was also low in these samples, with a median of 0.2 ng/μL (Table 3). Similarly, indicators of RNA quality suggested that RNA was very heavily degraded in this sample group, with a median RIN value of 1.4 and a median DV200 value of 19% (Table 3). These data indicate that conditions of sample collection/processing may have a profound impact on the quantity and quality of RNA derived from self‐taken samples.

**Table 3:** Median Nucleic Acid Quantity and Quality Measurements from Two Cohorts of Self‐taken samples.

Sample cohort	Nanodrop RNA (ng/µL)	Qubit RNA (ng/µL)	Qubit DNA (ng/µL)	RIN	DV200 (%)
SHA	**251** (29–982)	**83** (21–780)	**26** (1–179)	**3.4** (1.1–6.2)	**58** (10–95)
SCCB	**11** (1–36)	**2** (0–26)^a^	**0.2** (0–5.5)	**1.4** (1–2.5)^b^	**19** (5–79)

^a^ 8/19 samples had no detectable RNA.

^b^ 9/19 samples returned no RIN due to a lack of quantifiable features.

**Figure 5:**
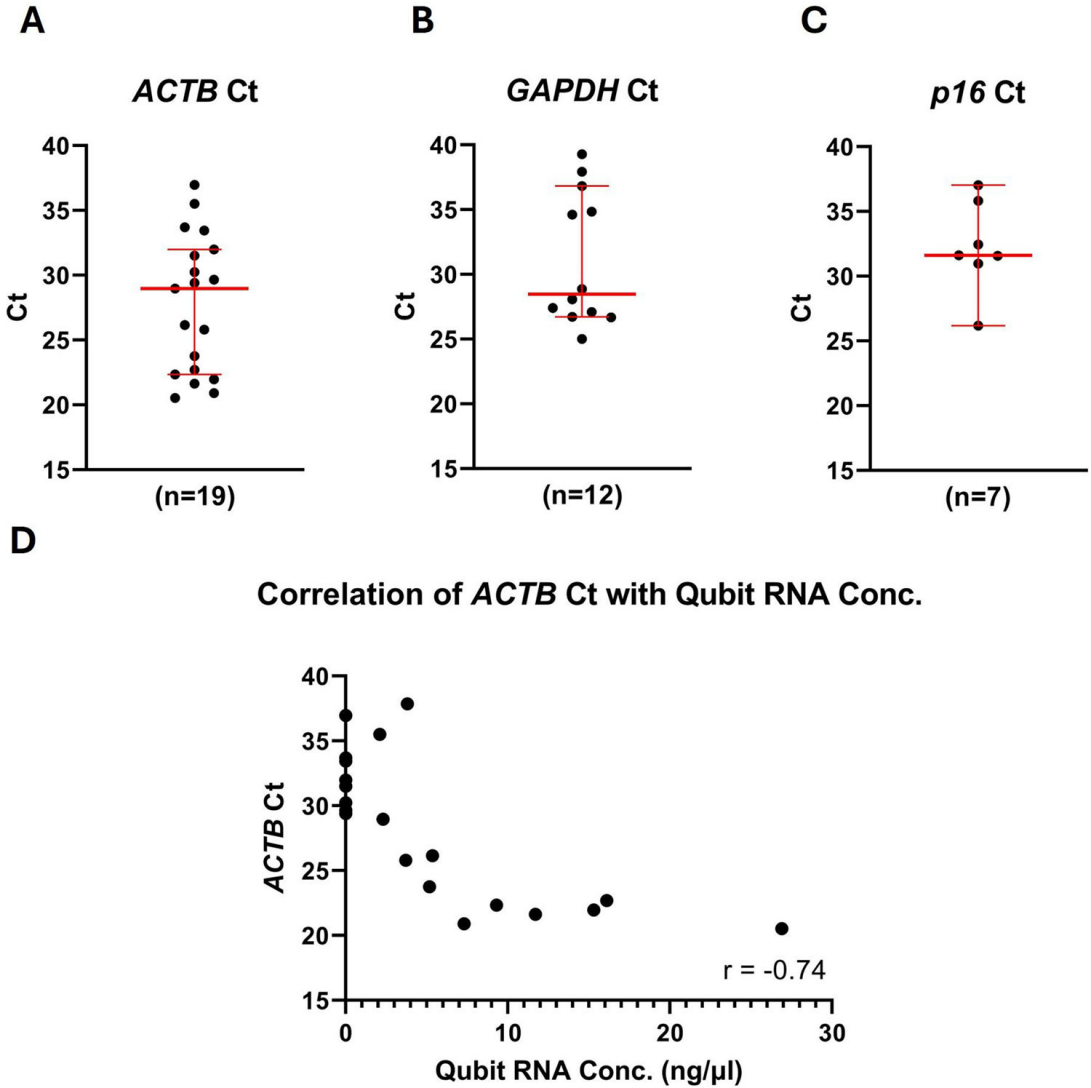
RT‐qPCR Testing of Self‐taken Swab Samples from the Swedish Cervical Cytology Biobank. Samples were analysed for expression of two reference genes; *ACTB* (A) and *GAPDH* (B), as well as a prospective biomarker, *p16* (C), to give a measure of suitability for mRNA expression testing. Each point represents an individual sample. *ACTB* was detected in all tested samples (*n* = 20), while *GAPDH* was detected in 12/20 samples and *p16* was detected in 7/20 samples. Ct = cycle threshold, low Ct = high expression of target. Red lines = median and 95% CIs. The correlation between RNA concentration measured by Qubit and *ACTB* Ct was also assessed (D). Each point represents the Ct for *ACTB* in an individual sample. r = Spearman's correlation coefficient.

Although several samples from the SCCB had no detectable RNA by Qubit testing, *ACTB* was detected in all samples by RT‐qPCR (Figure 5A), while *GAPDH* was detected in only 12/19 samples (Figure 5B). As expected, based on detection of the reference genes, *p16* levels were below detection in most samples from the SCCB cohort. Only 7/19 samples showed replicable detection of *p16* (Figure 5C). As control reverse transcriptase‐negative reactions from the same sample set showed no consistent amplification of the targets, these results are not due to amplification of residual gDNA. This highlights the importance of optimal sample collection and processing, as well as the selection of genes expressed at an appropriate level to ensure good quality quantifiable data.

As *ACTB* was the only reliably detected reference gene in this sample set, the correlation between *ACTB* Ct and RNA concentration was investigated. In this sample cohort, there was a significant negative correlation between the two variables, meaning higher RNA concentration corresponded to lower Ct values (Figure 5D). This indicates that while RNA concentration may not be a good overall predictor of reference gene Ct, very low concentrations of RNA are indicative of samples which are unsuitable for analysis by RT‐qPCR.

## 4 Discussion

Biobanks are a crucial resource for the development of novel diagnostic tests, including in the field of HPV research [28]. We investigated the suitability of biobanked self‐taken vaginal swab samples for use in PCR‐based RNA biomarker testing. RNA from the SHA samples was degraded but was still suitable for RT‐qPCR, similar to RNA isolated from clinician‐taken LBC samples [22]. Due to degradation, these samples are not recommended to be used for next‐generation sequencing, which has previously been utilised for RNA biomarker discovery using fresh‐frozen cervical cancer biopsies [29]. Although expression of reference genes *ACTB* and *GAPDH* was detected in all samples, *p16* expression was only detected in 82.1% of samples suggesting an average lower level of expression than the reference genes. For biomarker development it will be important to select genes which are expressed at an adequate level for detection by RT‐qPCR.

In this study, all samples underwent two rounds of DNase treatment, firstly during RNA extraction and secondly before cDNA synthesis. This protocol was sufficient to reduce gDNA to undetectable levels by RT‐qPCR in most samples. However, 11.6% of samples were highly contaminated with gDNA following RNA extraction. This contamination impacted RNA quality score assessments and subsequent amplification, as further DNase treatment before cDNA synthesis did not prevent false‐positive results by RT‐qPCR. A proportion of samples were contaminated following both initial RNA extraction and extraction following a year of storage, indicating that this effect may be partially due to a high concentration of DNA in the initial samples. DNA in the samples could form tertiary structures with lipids and/or proteins which could inhibit enzyme access. However, some samples were contaminated in the first set of extractions but not the second (and vice‐versa), suggesting other factors also contribute. Cell aggregates that were unable to be resuspended through pipetting were present in some samples. Long term storage could either cause increased solubility of these aggregates, releasing more DNA into solution, or cause consolidation of the aggregates due to evaporation trapping DNA. The variable presence of these aggregates, and the possibility of DNase inhibitors may respectively lead to higher concentrations of DNA or decreased DNase activity in different extractions from the same sample. Investigation of engineered DNase enzymes which more efficiently degrade gDNA would be beneficial for minimising this issue in further studies. In addition, primers could be designed to bind to and amplify across adjacent exons, therefore inhibiting amplification of gDNA. Together with DNase treatment, both during RNA extraction and in‐solution before cDNA synthesis, this should obviate the risk of false positive results.

The comparative performance of self‐taken samples relative to clinician‐taken samples in RT‐qPCR is an important consideration for the development of future mRNA biomarker‐based tests [30]. In a previous study by our group using the same extraction protocol, the average RNA concentration in clinician‐taken LBC samples was 76 ng/μL [22], which is lower than the average of 274 ng/μL reported for self‐taken samples in this investigation. However, a direct comparison of these results is impossible due to the lack of Qubit testing in the LBC study, meaning only the less reliable Nanodrop readings can be compared, and the lack of high‐grade cervical disease samples in the present study, which have previously been reported to yield less RNA [22]. Future studies of matched clinician‐taken and self‐taken samples would be useful in assessing the comparative performance of self‐taken samples in mRNA biomarker analysis.

Several studies have investigated the contribution of various factors involved in sample collection and storage to the final yield of RNA in clinician‐taken samples [31, 32]. An important finding of the current study was that storage of swab samples at −70°C for 1 year did not result in a decrease in the quality or quantity of RNA obtained. Instead, median Qubit RNA concentration and DV200 values were significantly increased in stored samples, which may be due to improved processing efficiency and the use of a different reagent batch during the second round of RNA extractions. These results suggest that storage of these samples for long periods of time does not preclude their use in the development of new clinical assays. We found significant differences in RNA yield between samples from the SHA and the SCCB. Although both sample cohorts were collected around the same period (2020–2021, 2020–2023) and both were predominantly mailed‐in samples, the two cohorts differed in many other respects. These include (1) collection device (Aptima multitest swab *vs.* cobas dual swab), (2) storage buffer and initial volume (Specimen Transport Media, 2.9 mL *vs.* cobas PCR Media, 4.3 mL), (3) sample storage temperature (initially −20°C, then −80°C *vs.* −25°C) and (4) extraction volume per sample (400 µL *vs.* 100 µL). Accounting for differences in initial and processed volumes between the two cohorts, a median RNA conc. of ~36 ng/µL would be expected for SCCB samples (compared to an observed median of 3 ng/µL). Therefore, while differences in volume were likely a major contributor to the variation between the cohorts, they do not fully explain the lower RNA concentration obtained from the SCCB samples. While Aptima tests are optimised for detection of hrHPV mRNA, the cobas system is based on detection of hrHPV DNA, which may contribute to the differences in RNA quantity/quality observed between the cohorts. Further studies involving prospective recruitment of patients are required to comprehensively evaluate the major determinants of RNA quality and quantity in self‐taken samples, ensuring that future biobank samples are suitable for this form of analysis.

## 5 Conclusions

Overall, this study shows that self‐taken samples can be used in RT‐qPCR and that mRNA‐based biomarker testing is feasible, laying the groundwork for new mRNA‐based biomarker tests. The data demonstrate the importance of optimising sample collection and storage protocols in the future to allow the use of self‐taken samples for this purpose.

## Author Contributions

Conceptualisation: K.C., S.V.G. Formal Analysis: H.S., D.M., W.R. Investigation: H.S., D.M., W.R. Resources: H.M., L.S.A.M., K.C. Writing ‐ Original Draft: H.S., S.V.G. Visualisation: H.S. Supervision: K.C., S.V.G. Project Administration: A.S., H.M., K.C., S.V.G. Funding Acquisition: K.C., S.V.G.

## Ethics Statement

The Scottish HPV Archive (SHA) is a biobank that comes under the auspices of NHS Lothian National Research for Scotland bioresource (REC 25/ES/0030). Self‐taken vaginal swab samples from the SHA were obtained through an application to the SHA Steering Group (application reference SA‐0069). Collection of Swedish Cervical Cytology Biobank (SCCB) samples and use for biological research was approved by the Regional Ethical Review Board in Stockholm (decision number 2014/1242‐31/4).

## Conflicts of Interest

Kate Cuschieri's laboratory has received research funding or gratis consumables to support research from the following commercial entities in the last 3 years: Cepheid, Euroimmun, GeneFirst, SelfScreen, Hiantis, Seegene, Roche, Hologic, Barinthus Biotherapeutics PLC & Daye. Kate Cuschieri has attended advisory board meetings for Hologic and Barinthus Biotherapeutics PLC, for the former, UK travel has been paid. The authors have no other competing interests or relevant affiliations with any organization or entity with the subject matter or materials discussed in the manuscript apart from those disclosed.

## Supporting information


**Supporting Figure S1:** Examples of gDNA Contamination in Select Samples following RNA Extraction. All sample lanes show DNA contamination except for lane 8, which is included as an example of uncontaminated RNA from a different self‐taken swab. gDNA in the other lanes is likely partially degraded through DNase treatment during RNA extraction. EL = Electronic RNA ladder.

supmat.

## Data Availability

All underlying data for the graphs in this study are available upon request.
