# Supplementary figures and images for: Viability of Self‐Taken Vaginal Swab Samples for RNA‐Based Biomarker Analysis in Cervical Disease
Source: J Med Virol. 2025 Dec 8;97(12):e70737. doi: 10.1002/jmv.70737 (PMC12683698; doi:10.1002/jmv.70737)

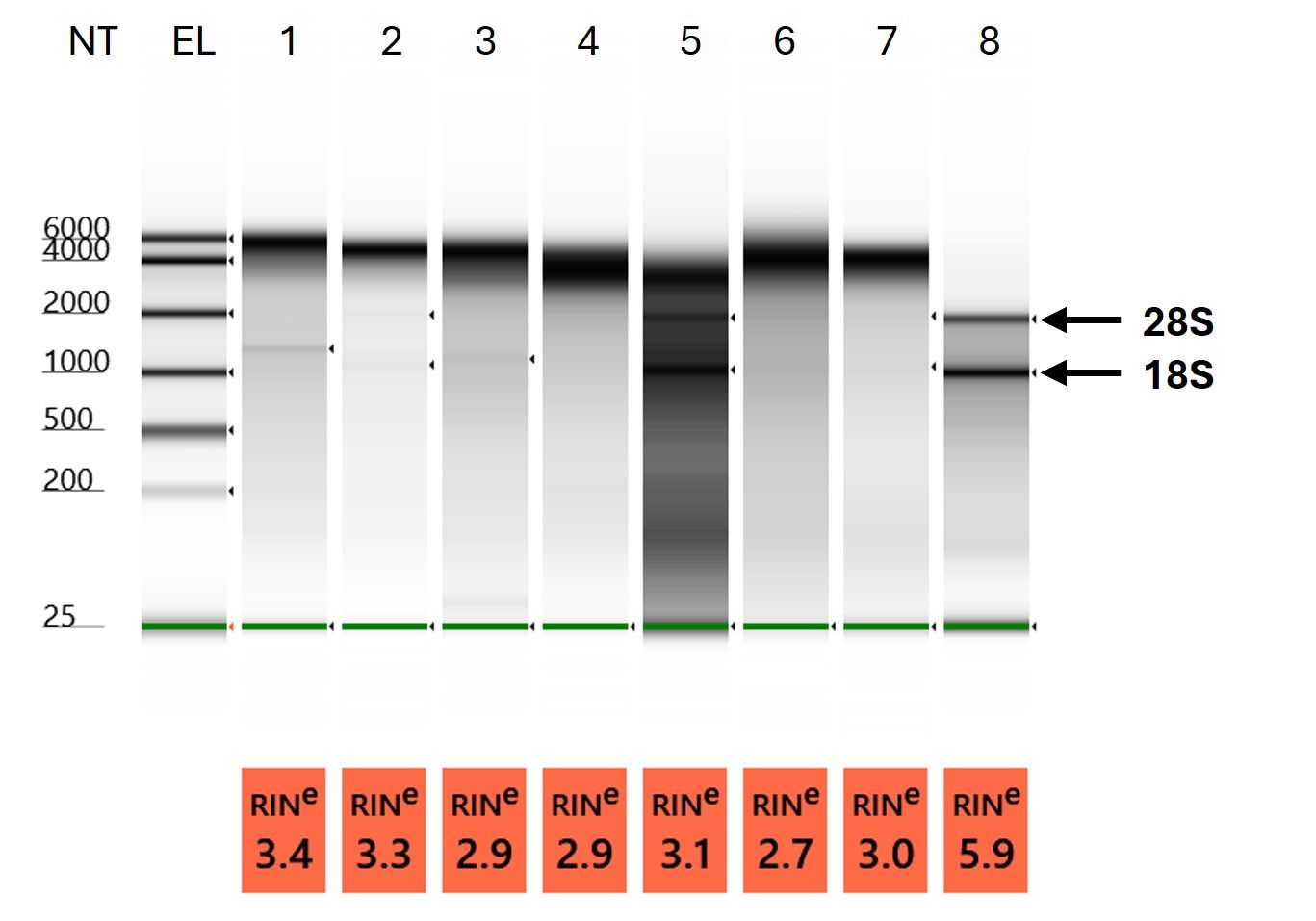

Supplement: Supplementary file 1 — Supporting Figure S1: Examples of gDNA Contamination in Select Samples following RNA Extraction. All sample lanes show DNA contamination except for lane 8, which is included as an example of uncontaminated RNA from a different self‐taken swab. gDNA in the other lanes is likely partially degraded through DNase treatment during RNA extraction. EL = Electronic RNA ladder. [file JMV-97-e70737-s002.jpg]
