# Supplementary material for: Viability of Self‐Taken Vaginal Swab Samples for RNA‐Based Biomarker Analysis in Cervical Disease
Source: J Med Virol. 2025 Dec 8;97(12):e70737. doi: 10.1002/jmv.70737 (PMC12683698; doi:10.1002/jmv.70737)
Supplement: Supplementary file 2 — supmat. [file JMV-97-e70737-s001.docx]

**Supplementary Table S1: Allplex HPV HR Detection Results for HPV-positive Samples**

| **hrHPV Genotype** | **Number of Positive Samples by Disease Group** | |
| --- | --- | --- |
|  | **No Disease (n**▒=▒**27)** | **CIN1 (n**▒=▒**18)** |
| HPV16 | 3 (11.1%) | 4(22.2%) |
| HPV18 | 0 | 0 |
| HPV45 | 1(3.7%) | 1(5.6%) |
| HPV33 | 1(3.7%) | 1(5.6%) |
| HPV58 | 1(3.7%) | 2(11.1%) |
| HPV31 | 3(11.1%) | 1(5.6%) |
| HPV52 | 2(7.4%) | 2(11.1%) |
| HPV35 | 2(7.4%) | 0 |
| HPV59 | 0 | 0 |
| HPV39 | 1(3.7%) | 1(5.6%) |
| HPV68 | 7(25.9%) | 3(16.7%) |
| HPV51 | 1(3.7%) | 2(11.1%) |
| HPV56 | 3(11.1%) | 1(5.6%) |
| HPV66 | 1(3.7%) | 0 |

**Supplementary Table S2: Amplification of *ACTB* and *GAPDH* from material in excised gel fragments.** Three RNA samples with visible contamination on Tapestation were run on a gel and contaminating fragments were excised and purified for use in qPCR. Ct = cycle threshold.

| **Sample No.** | ***ACTB* Ct** | ***GAPDH* CT** |
| --- | --- | --- |
| 1 | 26.0 | 24.2 |
| 2 | 28.4 | 25.9 |
| 3 | 25.4 | 23.3 |
